# The effects of intermittent fasting diet alone or in combination with probiotic supplementation in comparison with calorie-restricted diet on metabolic and hormonal profile in patients with polycystic ovary syndrome: study protocol for a randomized clinical trial

**DOI:** 10.1186/s13063-023-07691-5

**Published:** 2023-10-25

**Authors:** Sepide Talebi, Sakineh Shab-Bidar, Hamed Mohammadi, Ashraf Moini, Kurosh Djafarian

**Affiliations:** 1 Department of Clinical Nutrition, School of Nutritional Sciences and Dietetics, Tehran University of Medical Sciences, Tehran, Iran; 2 Students’ Scientific Research Center (SSRC), Tehran University of Medical Sciences, Tehran, Iran; 3 Department of Community Nutrition, School of Nutritional Sciences and Dietetics, Tehran University of Medical Sciences, Tehran, Iran; 4 Breast Disease Research Center (BDRC), Tehran University of Medical Sciences, Tehran, Iran; 5 Department of Obstetrics and Gynecology, Arash Women’s Hospital, Tehran University of Medical Sciences, Tehran, Iran; 6 Department of Endocrinology and Female Infertility, Reproductive Biomedicine Research Center, Royan Institute for Reproductive Biomedicine, ACECR, Tehran, Iran

**Keywords:** Intermittent fasting, Probiotics, Polycystic ovary syndrome

## Abstract

**Introduction:**

Polycystic ovary syndrome (PCOS) is one of the most common endocrine disorders in females characterized by ovulatory dysfunction, hyperandrogenism, and other metabolic disorders. Both intermittent fasting and specific probiotics have been suggested to help improve patients with PCOS through changes in gut microbial composition, circadian clock, and metabolic regulation. Therefore, the present study aims to investigate the effects of intermittent fasting alone or in combination with probiotic supplementation compared to the calorie-restricted (CR) diet on anthropometric measures, metabolic status, inflammation, and oxidative stress in women with PCOS.

**Methods:**

We will carry out a randomized clinical trial for 8 weeks. Participants will be randomly assigned (1:1:1) to one of the three groups: (1) a 14:10 early time-restricted feeding (TRF) diet with probiotic supplementation (*n* = 30); (2) a 14:10 early TRF diet with placebo supplementation (*n* = 30); (3) a CR diet (energy-restricted 25% of required calories) with placebo supplementation as a control group (*n* = 30). The primary outcomes will be changes in body weight and insulin resistance. However, glycemic control, lipid profile, metabolic parameters, sex hormone-binding globulin, dehydroepiandrosterone, anti-Mullerian hormone, free androgen index, hirsutism, acne, antioxidant and oxidant status, inflammation, anthropometric measures, mental health, sleep quality, appetite, eating behavior, food craving, and blood pressure are secondary outcomes. All outcomes of this study will be evaluated in pre- and post-intervention.

**Discussion:**

We hypothesized that 10-h TRE administered alone or in combination with probiotic supplementation to overweight and obese PCOS subjects would lead to weight loss and improved metabolic, hormonal, inflammatory, and antioxidant markers compared to control subjects following a standard 3-meal-per-day CR diet.

**Ethical aspects:**

The current trial received approval from the Medical Ethics Committee of Tehran University of Medical Sciences, Tehran, Iran (IR.TUMS.MEDICNE.REC.1401.425).

**Trial registration:**

Iranian Registry of Clinical Trials IRCT20121110011421N5. Registered on 3 October 2022.

**Supplementary Information:**

The online version contains supplementary material available at 10.1186/s13063-023-07691-5.

## Introduction

Polycystic ovary syndrome (PCOS), one of the most common endocrine disorders in females, approximately affects 7 to 12% of women of reproductive age [1]. High androgen levels, ovulation disorder, and the presence of polycystic ovaries are considered diagnostic criteria for PCOS. Also, an increase in testosterone, luteinizing hormone (LH), and anti-Müllerian hormone (AMH) and a decrease in the secretion of follicle-stimulating hormone (FSH) contribute to the development of PCOS [2]. A wide range of reproductive abnormalities including menstrual disorders, infertility, hirsutism, and hyperandrogenism are represented in PCOS. Beyond hormonal disorders, obesity, insulin resistance, and chronic inflammation that increase the risk of metabolic syndrome, type 2 diabetes, and cardiovascular diseases are often associated with PCOS [3].

Recently, dietary interventions have been taken into consideration for the management of PCOS. Because up to 60% of PCOS women are overweight or obese, evidence-based international guidelines emphasize the importance of diet and recommend dietary and exercise interventions as the first line of management for this disorder [4]. To date, several diets including low-glycemic index diet [5], DASH diet [6], Mediterranean diet [7], low-carbohydrate diet [8], and ketogenic diet [9] have been proposed for the management of PCOS. However, there is a knowledge gap to suggest the best particular dietary intervention for improving PCOS health outcomes [4].

Intermittent fasting (IF) is a promising alternative, which has been shown to improve cardiometabolic status while adhering to weight loss goals [10]. IF is the practice of alternating between eating and fasting. IF is a general term for three different types of diets: alternate-day fasting (ADF), the 5:2 diet, and time-restricted feeding (TRF) [11]. Among the IF diets, the TRF diet is a lifestyle intervention that limited the duration of food intake to a fixed number of hours, without calorie counting or dietary recommendations [12]. Besides, past considerations recommend that TRE is attainable and accessible to upgrade adherence [13, 14]. IF diets could be beneficial in reducing body weight, improving insulin resistance, and reducing inflammation by affecting the circadian rhythm, intestinal microbial composition, and regulating metabolism [15]. Since disturbance in the circadian rhythm is related to insulin resistance, excess androgen production, increased levels of AMH, and apoptosis of granulosa cells, IF diets especially the TRF diet might alleviate PCOS by improving circadian rhythm [16–20]. Also, IF diets could ameliorate PCOS by decreasing insulin resistance through stimulation of AMP-activated protein kinase (AMPK) [21].

On the other hand, the microbiological hypothesis proposed that the microbial composition of the gut plays a crucial role in the pathogenesis of PCOS. Recent studies have demonstrated that dysbiosis of gut microbiota is related to sex hormone levels and ovarian morphological changes [22, 23]. Also, an imbalance in the gut microbial composition is associated with obesity, insulin resistance, and inflammation [24]. Probiotics could be used as a probable treatment for PCOS by changing gut microbiota, reducing insulin resistance, and improving inflammatory, antioxidant, and hormonal status [25]. Certain species of *Lactobacillus* and *Bifidobacterium* have been proposed as the most effective species for regulating sex hormones and metabolic parameters [26, 27]. Lactobacillus species contain beta-glucuronidase enzymes, allowing them to break down estrogens in the gut [28]. Moreover, *Lactobacillus* species resulted in the production of short-chain fatty acid metabolites (SCFAs) that reduced insulin resistance and inflammation and modulated androgen levels [29]. It has been shown that lactobacillus transplantation could partially reduce androgen production as well as the abnormalities in the estrous cycle and ovarian morphology caused by letrozole in PCOS-like rats induced with letrozole [30]. *Lactobacillus rhamnosus* supplementation enhanced the effectiveness of weight loss therapy for obese women, as demonstrated by Hulston et al. [29]. According to reports, *Limosilactobacillus reuteri* regulates host lipid metabolism, insulin sensitivity, inflammatory status, and immune response [31, 32]. The *Limosilactobacillus reuteri* was shown to improve glucose tolerance in rats with constant darkness-induced PCOS in a previous study [33].

Several studies investigating the synergistic effect of probiotic supplementation with a low-calorie diet have reported profitable effects of them to improve health status [34–37]. A recent study has demonstrated that the administration of probiotics in addition to a calorie-restricted (CR) diet resulted in significant weight loss [37]. Therefore, probiotics were introduced as an innovative strategy to increase the effectiveness of CR diets for weight control [38]. To the best of our knowledge, no clinical trial has investigated the effect of IF diet combination with probiotics supplementation in patients with PCOS. Therefore, the present study aims to investigate the effects of IF alone or in combination with probiotic supplementation compared to the CR diet on anthropometric measures, metabolic status, inflammation, and oxidative stress in women with PCOS.

## Subjects and methods

### Study design

**Fig. 1:**
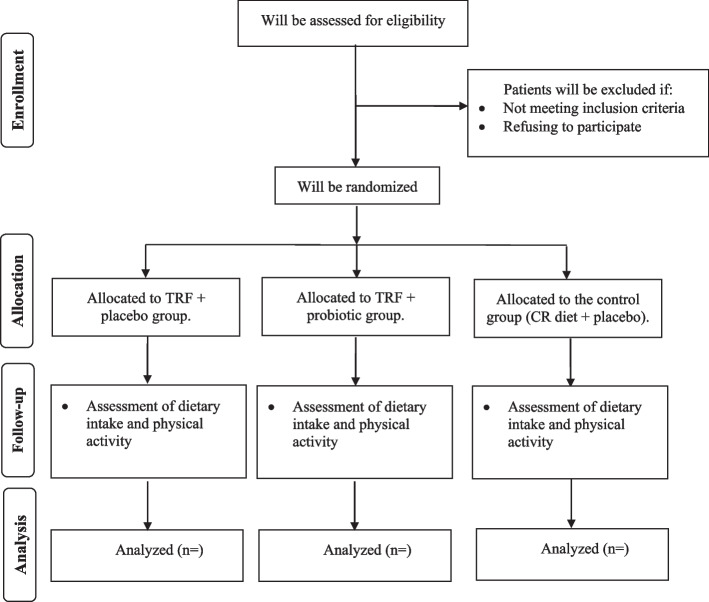
Summary of the patient flow diagram. TRF, time-restricted feeding; CR, calorie restricted

**Fig. 2:**
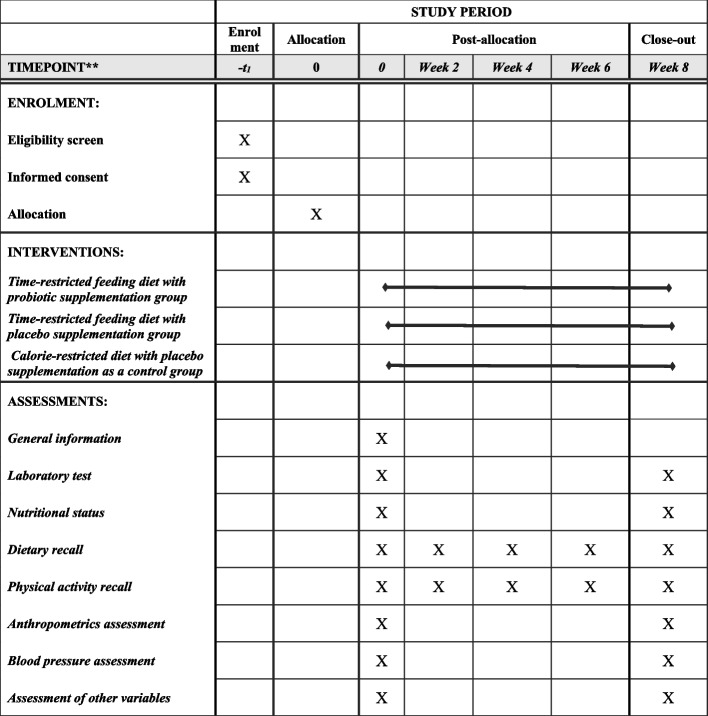
SPIRIT diagram of the recommended content for the schedule of enrolment, interventions, and assessments

The present placebo-controlled, parallel-arm, randomized, double-blind clinical trial examining the effects of IF diet alone or in combination with probiotic supplementation compared to the CR has been registered at the Iranian Registry of Clinical Trials (ID: IRCT20121110011421N5) on October 3, 2022. This trial will be performed at Arash Hospital, Tehran, Iran. Written informed consent will be obtained from participants before participation in the research project by researchers. We developed the study protocol based on the Standard Protocol Items: Recommendations for Interventional Trials (SPIRIT) 2013 checklist (Supplemental file 1). The timeline of the trial and study flow chart of enrolment, allocation, intervention, and assessment are presented in Table 1 and Fig. 1, respectively. A Standard Protocol Items: Recommendations for Interventional Trials (SPIRIT) diagram for the trial schedule is illustrated in Fig. 2. Any amendments to the present study protocol which have related to the safety and benefit of patients and the protocol deviations, unintentional alterations in study protocol that do not affect the subject’s rights, study’s risk or benefit, the integrity of data, and safety or welfare will require to be confirmed by Department of Clinical Nutrition and Medical Ethics Committee of Tehran University of Medical Sciences, Tehran, Iran, before the study conduction. Any alteration in the study protocol will be sent to the Trials journal (www.trialsjournal.biomedcentral.com).

**Table 1:** Timeline of the trial

Explanation of the trial activities	Time (months)																	
	1	2	3	4	5	6	7	8	9	10	11	12	13	14	15	`16	17	18
Material preparation	*	*	*	*														
Recruitment					*	*	*	*	*									
Clinical assessments at baseline					*	*	*	*	*									
Nutritional assessments at baseline					*	*	*	*	*									
Biochemical assessments at baseline					*	*	*	*	*									
Intervention										*	*	*	*	*				
Clinical assessments after intervention										*	*	*	*	*				
Nutritional assessments after intervention										*	*	*	*	*				
Biochemical assessments after intervention										*	*	*	*	*				
Data analysis															*	*		
Writing the final report of the trial																	*	*
The expected time	*	*	*	*	*	*	*	*	*	*	*	*	*	*	*	*	*	*

### Study setting

Subjects’ recruitment process will be advertised through the presence of patients at Arash hospital for treatment and diagnosis of diseases as well as through flyers distributed at women’s clinic centers in Tehran, affiliated with Tehran University of Medical Sciences, the province’s main center for treatment, prevention, research, and services. Participants will be selected from PCOS patients who are newly diagnosed by an expert gynecologist using Rotterdam criteria [39]. Candidates who are interested will be invited to the screening process.

### Study participants and enrollment

PCOS patients will be recruited from the clients of Arash Hospital by two independent researchers. An adept gynecologist with sufficient expertise in the diagnosis of PCOS will diagnose patients with PCOS based on the Rotterdam criteria. Patients who satisfy the following inclusion criteria will be eligible to participate in this research project.

### Inclusion criteria

The inclusion criteria are considered as follows: individuals aged between 18 and 40 years, subjects whose BMI ranged between 25 and 35 kg/m^2^ and who definitely eat breakfast, diagnosis of PCOS by a specialist based on the Rotterdam criteria for the first time (new cases) without medical treatment, and having or using a Smartphone.

### Non-entry criteria

Applicants will not be eligible if they are pregnant or breastfeeding or menopause; are night shift workers; are using antibiotics for at least 3 months before the intervention; have history of smoking, alcohol, and drug abuse; have inability to fast due to overnight medication; are consuming fermented products and foods containing probiotics; are being on a special or prescribed diet for various reasons; or have diabetes mellitus, hypertension, liver and kidney disorders, cancer, acute or chronic infectious diseases, type 1 and 2 diabetes, severe gastrointestinal diseases, Cushing’s disease, adrenal disease, acromegaly, gigantism, and an eating disorder.

### Exclusion criteria

Participants will be excluded if they had an antibiotic intake, allergic reaction to the supplement, getting infected with COVID-19, low adherence of patients to the intervention, or taking any medication affecting weight, appetite, hormonal status, carbohydrate, or lipid metabolism during the study.

### Study interventions

Participants will be randomly assigned (1:1:1) to one of the three groups for 8 weeks: (1) a 14:10 early TRF diet (eating ad libitum only between 8:00 AM and 6:00 PM) with probiotic supplementation; (2) an 14:10 early TRF diet (eating ad libitum only between 8:00 AM and 6:00 PM) with placebo supplementation; (3) a CR diet (energy-restricted 25% of required calories) with placebo supplementation as a control group.

During the TRF period, subjects are instructed to eat freely from 8:00 AM to 6:00 PM daily (for 10 h). While from 6:00 PM to 8:00 AM the next day (14 h), people will only be allowed to drink water, tea, and black coffee without sugar or any calorie-free drink. In addition, individuals will be suggested not to alter the composition of their usual diets throughout the TRF intervention. Furthermore, PCOS patients will be asked to consume a small handful of nuts consisting of 200 kilocalories of mixed nuts (18 g of fat, 5 g of protein, and 4 g of carbohydrates) during fasting if they experience drops in energy or severe headache [40].

In the CR diet of the control group, the daily calories will be calculated by a dietitian. Based on the Harris-Benedict formula, the basal metabolic rate (BMR) of patients will be calculated individually [41]. Then, the total daily energy needed by each person is calculated based on his physical activity and considering the thermogenic effect of food (TEF). Finally, 25% of the required calories [42] will be reduced. Subsequently, this daily caloric requirement will be divided into six-meal menus including 3 main meals and 3 snacks with a macronutrient ratio of 55% carbohydrates, 30% fat, and 15% protein. Based on this, a food menu will be prepared, and the necessary alternatives will be taught to the people of the control group. All participants will also take a probiotic or placebo supplement after breakfast with a glass of water.

### Randomization and blinding

A stratified permuted block randomization (a fixed block size of *n* = 6) was done using computer generated random numbers by the project coordinator. In this method, eligible patients who meet the study entry criteria are selected and then randomly allocated with stratification by BMI (between 25 and 30 and 30 and 35 kg/m^2^). People will be allocated to one of the three groups, IF diet with a probiotic supplement, IF diet with a placebo, and CR diet with a placebo. Random allocation will be based on a random number list; the letters A, B, and C will be assigned to an equal number of a random number list.

Placebo and probiotic supplements will be produced by TakGen Zist Pharmaceutical Company (Tehran, Iran) and will be placed in the same packages with similar color, flavor, and taste. Then packages will be placed in paper boxes and receive a special code. Placebo and probiotic supplement codes will remain with the company until the end of the study for blind researchers, laboratory staff, outcome assessors, and patients. However, no blinding will do in terms of diet (fasting or CR). In other hand, we do not anticipate any requirement for unblinding but if required, the trial manager, data coordinator, implementation support facilitators, and care home managers will have access to group allocations and any unblinding will be reported.

### Supplement administration

Daily probiotic capsules contain 1 × 10^9^ colony-forming units (CFU) with two strains of *Lactobacillus rhamnosus* and *Lactobacillus reuteri*, as well as magnesium stearate and lactose as carriers. It also contains 130 mg of starch without bacteria in the placebo compound. We used the suggested dosage from a previous study in the treatment of vulvovaginal candidiasis patients due to the lack of evidence-based approaches regarding the appropriate dosage of this probiotic supplement in subjects with PCOS [43].

### Adherence to the intervention

All subjects will receive telephone calls per week and monthly meetings with a dietitian during the intervention to encourage adherence to the diet. We will send a daily reminder via instant messages that inform them when to stop eating and when they are allowed to eat. Furthermore, periodic meetings (once every 30 days) should be promoted for participants to share their experiences and receive support from physicians. Moreover, all people are given detailed written and oral instructions for each meal plan, and as mentioned, there is no difference between people participating in the fasting diet and the low-calorie diet. Monitoring of food programs is done through the daily interaction of the researcher and the participant in the project through phone conversations. If they do not attend more than two consecutive phone sessions or consume 3 inappropriate meals or fail to follow the diet instructions 3 times a week for more than 2 consecutive weeks is considered non-compliant patients [44, 45]. A 3-day 24-h dietary recall will be taken from the participants every 2 weeks to confirm compliance.

### Outcomes

#### Primary and secondary outcomes

The primary outcomes in this study will be body weight and insulin resistance. However, fasting blood glucose (FBS) levels, lipid profile, insulin levels, homeostatic model assessment for insulin resistance (HOMA-IR), HOMA for β-cell function (HOMA-β), quantitative insulin sensitivity check index (QUICKI), sex hormone-binding globulin (SHBG), dehydroepiandrosterone (DHEA), AMH, free androgen index (FAI), hirsutism, acne, total antioxidant capacity (TAC), total oxidant status (TOS), C-reactive protein (CRP), body mass index (BMI), mental health, sleep quality, appetite, eating behavior, food craving, energy intake, systolic, and diastolic blood pressure will be known as secondary outcomes. All outcomes of this study will be evaluated in pre-and post-intervention.

#### Measurements and assessments

##### Clinical assessment

General information of patients including age, level of education, occupation, marital status, duration of disease diagnosis, time to sleep and time to wake up (sleep time), alcohol consumption, smoking history, antioxidant supplements, herbal medicines, and history of various diseases was collected through a general questionnaire by interview method at baseline.

##### Anthropometric and blood pressure assessment

Body weight will be measured by fasting, without shoes, with minimal clothing, and using a digital scale (Seca, Hamburg, Germany) with a precision of 100 grams at baseline and 8 weeks during the intervention. The height of people will be measured to the nearest 0.5 cm while standing without shoes. BMI will be calculated using the formula of dividing weight in kilograms by the square of height in square meters. To measure blood pressure, patients were asked to rest for 10 min, then the measurement will be done using a mercury sphygmomanometer (Riester, Germany). The blood pressure of each person will be measured twice with a time interval of 10 min, in a sitting position. The average of these two measurements is considered as the patient's blood pressure.

##### Assessment of physical activity

Twenty-four-hour physical activity recall will be used to assess physical activity. Participants will fully record all their activities during the day in the first, second, fourth, sixth, and eighth weeks of the study. The start time, end time, and intensity of each type of activity are recorded in the table. In total, the duration of all your recorded activities should be about 24 h [46]. Changes in physical activity levels during the study will be considered a confounding factor for statistical analysis.

##### Nutritional assessments

Food intake will be assessed through the 24-h dietary recall (two working days and one weekend day). Twenty-four-hour dietary recall of the first day is completed at the same time as the first visit, and the record of the second and third days is completed by a trained questioner over the phone. Food notes will be taken from the participants every 2 weeks. The amount of consumption of all food and beverages is asked from the person based on household scales and recorded in the 24-h recall questionnaire. The 24-h recall questionnaires are reviewed by a nutrition expert after completion. Dietary data was analyzed by Nutritionist IV software (First Databank, San Bruno, CA, USA) modified for Iranian foods.

##### Biochemical assessment

Blood samples will be collected from the participants in the intervention on the first day and at the end of the intervention, observing 12–14 h of fasting. A 10-ml blood sample without any anticoagulant is centrifuged at a speed of 3500 rpm for 10 min. The serum separated from each person’s blood sample is transferred into sterile microtubes. The microtubes will be stored at − 80 °C.

##### Assessment of other variables

We will be used visual analogue scales [VAS] [47]; Depression, Anxiety and Stress Scale-21 Items (DASS-21) [48]; Pittsburgh Sleep Quality Index (PSQI) [49]; food cravings questionnaire [50]; the three-factor eating questionnaire-R21 [51]; and global acne grading system [52] to assess appetite sensations, mental health, sleep quality, food craving, eating behavior, and acne score of individuals, respectively. All these factors will be measured at onset and end of the study. The validity and reliability of the questionnaires have already been evaluated in Iran [53–58].

### Sample size calculation

By consideration of a type I error of 5% (*α* = 0.05), a power of 80%, according to the mean weight loss of 2.5 kg (a minimum clinically important difference) as the principal outcome and the standard deviation of 3.87 and 3.04 [59], the sample size was computed to be 25 for each group based on the two-sided t-test. Primary outcomes will be measured as a change from baseline. To compensate for an approximate drop-out rate of 5% during the study period, we elevate the final sample size to 30 subjects in each group. To calculate the required sample size Gigacalculator software was used.

$$n=\frac{{\left({z}_{1-\frac{a}{2}}+{z}_{1-\beta }\right)}^{2}\left({{s}_{1}}^{2}+{{s}_{2}}^{2}\right)}{{\Delta }^{2}}$$

### Data management and monitoring

In the baseline and post-intervention phases, data will be collected. CRFs (case report forms) will be used for recording participant data. Data entry and management will be done using an electronic database after CRFs are used for data collection. There will be two data collectors, and completed clinical raw data will be sent for approval to the project supervisor. Documentation is kept in a locked drawer, only accessible by the principal investigator. The forms contain an ID number unique to the participant and do not contain any information that can be used to identify the patient.

A clinical trial monitor occasionally supervises the study progress and ensures patient rights and well-being are safeguarded; the protocol, ethical requirements, standards, and regulations are being followed; the essential documentation is available; and collected data are accurate as there were recorded. One of the investigators will check the coding, security, and storage of data. In addition, she will evaluate data entry and data values twice. If any participant reports the occurrence of adverse events, more information is required to make a decision about excluding the participants from the trial. Unblinding is permissible in this situation based on the Medical Ethics Committee criteria. Ethical review of the data will be overseen by the Tehran University of Medical Sciences. Further, patients who discontinue the study or deviate from it will be provided with a list of all outcome data. Missing data will be imputed using modern methods for dropouts.

### Composition of the data monitoring committee

Participants are at minimal risk during the intervention and measurement protocols. Since this trial is low-risk, close monitoring by the principal investigator and an independent Safety Officer is part of the data safety monitoring plan. Any adverse event that exceeds the threshold and any serious adverse event will be promptly reported to the NIH and the COMIRB by the principal investigator and an independent Safety Officer. During the annual Safety Officer meeting, the Safety Officer will review the study coordinator’s reports to determine if any corrective action, trigger for an ad hoc review, or stopping rule violation should be reported to the study investigators, the COMIRB, and the sponsor.

### Statistical analysis

All analyses will be performed according to the Intention to Treat Analysis (ITT) method. Therefore, all participants who entered the study (regardless of whether they completed the 8-week study period) are included in the analysis. Missing data will be checked by multiple imputations. Statistical analysis will be conducted using the SPSS software (Version 24, SPSS Inc., and Chicago, IL, USA). The scatter diagram, histogram, and Kolmogorov–Smirnov test will be applied to examine the normality of the data. Normally distributed variables will be reported as the mean and standard deviation, while the median and interquartile range (IQR) will be used for reporting non-normally distributed variables. Moreover, we will use the chi-square test and Fisher’s exact test to compare categorical variables. Also, the independent sample *t*-test and Mann–Whitney U test will be applied to compare continuous variables between-group, whereas we will use paired sample *t*-test and Wilcoxon rank-sum test for within-group comparisons. To compare the differences in primary and secondary outcomes between the two study groups at the end of the trial and adjust the final findings for potential confounders, we will apply the mixed linear effect model. To correct for multiple comparisons and reduce alpha error, *q* values will be used using the Benjamin-Hochberg method. Based on this, significance was defined in *P* values less than 0.05 and *q* values less than 0.1. Cohen’s *d* will be calculated based on the mean and standard deviation. The effect sizes 0.20, 0.50, and 0.80, respectively for low, medium, and high therapeutic effects of the dietary intervention will be determined. No subgroup analyses are planned. However, no interim analyses are planned for this trial.

### Consent

When participants sign the consent form, they will be asked if they consent to their data being used in the event of withdrawal. Also, researchers will ask participants for permission to share relevant data with people from the participating universities or regulatory authorities. Biological specimens are not collected for storage in this trial.

## Discussion

PCOS is a complex endocrine disorder characterized by ovulatory dysfunction, hyperandrogenism, and other metabolic disorders [60]. Disturbance in the body’s circadian clock rhythm and the imbalance of intestinal microbial composition and increased insulin can play a pivotal role in the pathogenesis of PCOS [15]. In recent years, lifestyle modification, dietary interventions, and the modification of intestinal microbial have been touted for helping to improve patients with PCOS [4]. Human and animal studies showed that the IF diets may be a promising intervention strategy through the effect on the body’s circadian clock, intestinal microbial composition, and regulation of metabolism can help reduce body weight, increase fat oxidation, improve insulin resistance, and reduce inflammation [61]. One form of IF is TRF, which is more adherent than other forms of IF [13, 14]. Although most human clinical trials of TRF did not explicitly restrict caloric intake and/or recommend any dietary recommendations, participants in the freely consumed TRF group typically reduced their caloric intake by 7–22% [62, 63]. In other words, TRF can influence energy expenditure to obtain a negative caloric balance [14]. However, there is surprisingly little empirical data to support the notion that reducing or eliminating food after a certain time of night leads to lower daily energy intake or effective weight management in healthy adults [64]. TRF studies show that increasing the duration of daily fasting to periods of more than 12 h may bring more cardiometabolic benefits [65]. However, daily fasting for 16 h or more usually requires skipping a meal, which can reduce adherence or lead to poorer food choices [66]. For this reason, some studies used the TRF diet with 14 h of fasting to increase the adherence of participants [62, 63, 66–69]. Emerging evidence has proposed that an early TRF diet (food consumption in the early hours of the day) compared to a delayed TRF (food consumption in the last hours of the day) was effective for improved glucose tolerance, body weight control, and cardiometabolic effects [70, 71]. Indeed, the mechanism of favorable effects of early TRF is related to central and peripheral circadian oscillators. So that eating between 8:00 am and 6:00 pm optimizes the functioning of the organs and the environment involved in metabolism, and because the time of eating corresponds to the fluctuations of metabolic hormones, it can prevent the development of metabolic diseases and diabetes [71].

Patients with PCOS have significant changes in the composition of the gut microbiota. This change was associated with a decrease in α diversity and changes in β diversity [22]. There is growing evidence that gut microbiota dysbiosis is related to sex hormone levels, estrous cycles, and ovarian morphological changes [23]. Based on a large amount of data, the administration of probiotics protects the intestinal barrier, consequent in the formation of short-chain fatty acid metabolites (SCFAs) [29]. It can show unique effects for reducing insulin resistance, inflammation, and modulating androgen levels [25]. *Lactobacillus rhamnosus* supplementation enhanced the effectiveness of weight loss therapy for obese women, as demonstrated by Hulston et al. [29]. Also, *Lactobacillus reuteri* was shown to improve glucose tolerance in rats with constant darkness-induced PCOS in a previous study [33]. In addition, clinical trials have shown that *Lactobacillus rhamnosus* and *roteri* can play a positive role in restoring the vaginal microecology and treating bacterial vaginosis. So, correcting the microbial condition of the vagina by improving the menstrual cycle and AMH level leads to improvement in fertility [72]. Probiotics may have additional benefits to increase efficacy for weight control and improve cardiometabolic status when combined with an IF intervention or other CR diet [36, 73].

We hypothesized that 10-h TRE administered alone or in combination with probiotic supplementation to overweight and obese PCOS subjects would lead to weight loss and improved metabolic, hormonal, inflammatory, and antioxidant markers compared to control subjects following a standard 3-meal-per-day CR diet. To the best of our knowledge, no clinical trial has investigated the effect of IF diet combination with probiotics supplementation in patients with PCOS. Therefore, the present study aims to investigate the effects of IF alone or in combination with probiotic supplementation compared to the CR diet on anthropometric measures, metabolic status, inflammation, and oxidative stress in women with PCOS. If successful, nutritionists in clinical environments may recommend a new eating pattern (TRF) alone or along with probiotic supplements for patients with PCOS to treat this common disorder.

## Trial status

Iranian Registry of Clinical Trials IRCT20121110011421N5. Registered on 3 October 2022. The recruitment started on 23 October 2022 and will be almost finished on 23 September 2023.

### Supplementary Information

**Additional file 1.** SPIRIT 2013 Checklist.

## Data Availability

The data from future studies will be published. Corresponding authors will be able to access raw data upon request.

## Abbreviations

PCOS: Polycystic ovary syndrome
AMH: Anti-Müllerian hormone
FSH: Follicle-stimulating hormone
VLCD: Very low-calorie diets
IF: Intermittent fasting
TRF: Time-restricted feeding
AMPK: AMP-activated protein kinase
SCFA: Short-chain fatty acids
CR: Calorie-restricted
CFU: Colony-forming unit
FBS: Fasting blood glucose
HOMA-IR: Homeostatic model assessment for insulin resistance
QUICKI: Quantitative insulin sensitivity check index
SHBG: Sex hormone-binding globulin
DHEA: Dehydroepiandrosterone
FAI: Free androgen index
TAC: Total antioxidant capacity
TOS: Total oxidant status
CRP: C-reactive protein
BMI: Body mass index
PSQI: Pittsburgh Sleep Quality Index
